# Low‐intensity focused ultrasound, a novel approach to epilepsy treatment in developing countries

**DOI:** 10.1002/brb3.2852

**Published:** 2022-12-21

**Authors:** Sadaf Afif, Syeda Tayyaba Rehan, Hassan ul Hussain, Md. Saiful Islam

**Affiliations:** ^1^ Touro College of Osteopathic Medicine New York New York USA; ^2^ Department of Medicine Dow University of Health Sciences Karachi Pakistan; ^3^ Department of Public Health and Informatics Jahangirnagar University Savar Dhaka Bangladesh; ^4^ Centre for Advanced Research Excellence in Public Health Savar Dhaka Bangladesh

**Keywords:** developing countries, epilepsy, surgery, treatment, ultrasound

## Abstract

Approximately 80% of patients with epilepsy reside in poor resource settings. Despite the continued advancements and development of new treatment approaches, epilepsy remains a major health problem in developing countries. Consistent findings of epidemiologic studies reflect that both prevalence and treatment gap are higher in the developing world. The objective of this short review was to evaluate current treatment options and low‐intensity, pulsed‐focused ultrasound (FUS) as a potential new treatment option for epilepsy. Although some of the patients could be candidates for surgery, many factors, including poor health‐care infrastructure, socioeconomic status, risks and complications associated with the surgery, and patients’ preferences and attitudes toward the surgical procedure, limit the adherence to get surgical therapies. Low‐intensity FUS, a novel and noninvasive therapeutic approach, has the potential to be approved by regulatory bodies and added to the list of standard treatment options for epilepsy. Improved understanding of epilepsy's prevalence and incidence in developing worlds, identification of potential new therapeutic options, and their evaluation through continuous studies and clinical trials are needed to reduce the burden of epilepsy and the treatment gap.

## Graphical abstract

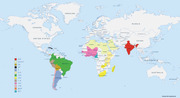

## 1 BACKGROUND

**FIGURE 1:**
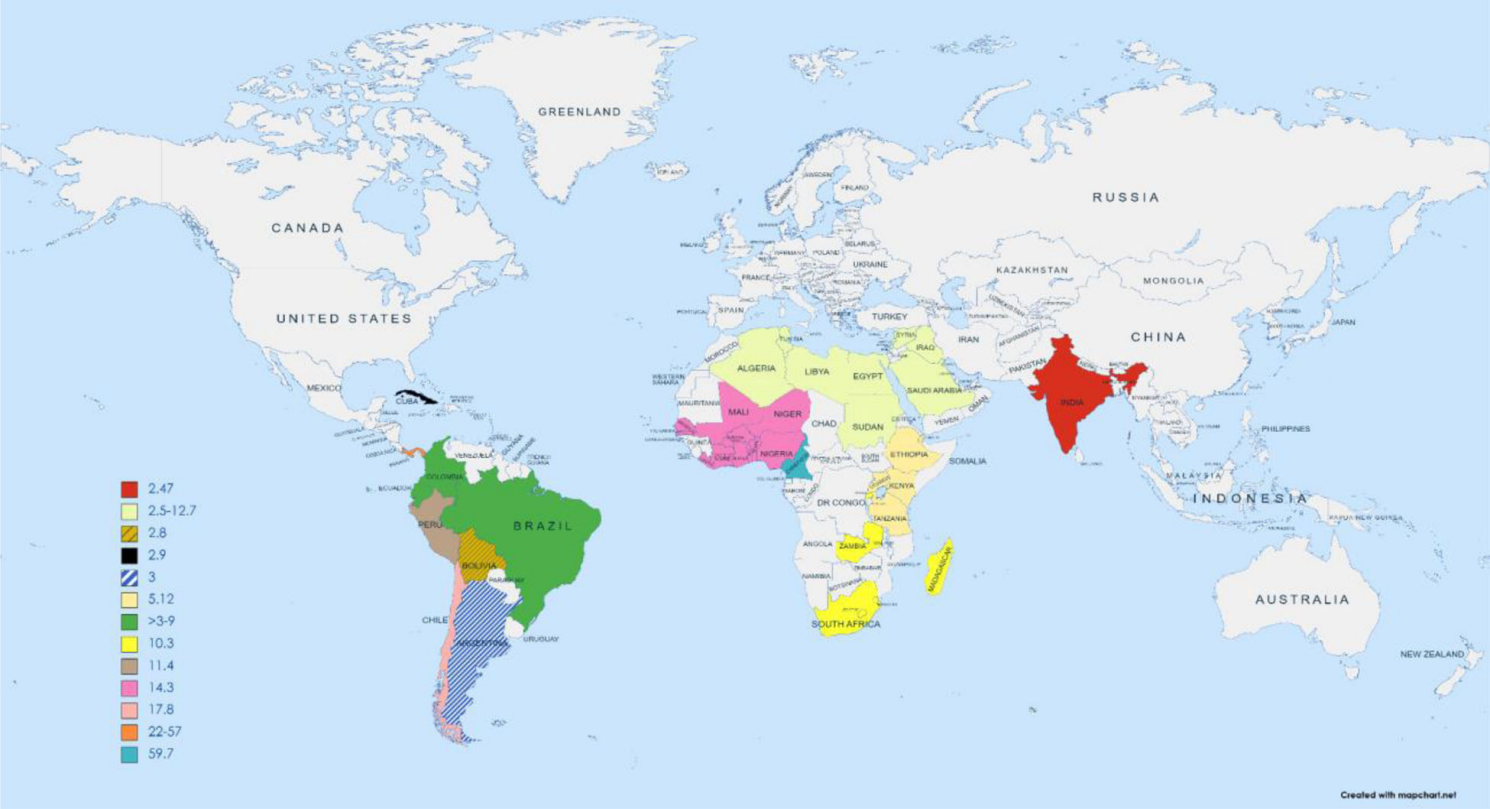
Regional map showing rates of epilepsy prevalence per 1000 people amongst developing and lower‐ and middle‐income countries (Idris et al., 2021; Ba‐Diop et al., 2014; Alva‐Díaz et al., 2021; Senanayake & Román, 1993)

Epilepsy is one of the most common chronic noncommunicable neurological disorders, affecting approximately 50 million people worldwide (World Health Organization, 2022). There is a significant difference in regional, national, and global incidence and prevalence of epilepsy depending on various characteristics such as sociodemographic characteristics of the population at risk, variations in medical and transport infrastructures, and so on. There are also variations depending on available population‐based studies, methodology differences, and data collection timeframe. Although incidence rates of epilepsy seem to be decreasing in high‐income countries (HICs), it remains a major health concern in lower‐ and middle‐income countries (LMIC) (Wahab, 2010). It is estimated that 80% of epilepsy affected individuals live in the LMICs (Figure 1) (Wahab, 2010). According to a systematic review and meta‐analysis of international studies by Beghi (2020), the pooled incidence rate of epilepsy was estimated to be around 61.4 cases per 100,000 persons‐year and is higher in LMICs compared to HICs (139.0 vs. 48.9 persons per 100,000) (Beghi, 2020). Moreover, the lifetime prevalence of epilepsy was reported to be 7.60, whereas the point prevalence of active epilepsy was estimated to be 6.38 per 10,000 persons. The overall lifetime (8.75 vs. 5.18 per 1000 persons) and median point prevalence (6.68 vs. 5.49) were also higher in the LMICs compared to the HICs (Beghi, 2020).

Several underlying pathophysiological mechanisms and risk factors are associated with epilepsy. These risk factors include but are not limited to perinatal and birth injuries, congenital disorders, acquired traumatic brain injuries, risk of comorbid health conditions such as strokes, depression, migraine, neurodegenerative, neurocysticercosis, and other infectious diseases (Seidenberg et al., 2009; World Health Organization, 2022). Comorbid physical and neuropsychiatric conditions are highly common in patients with epilepsy (PWE) with several associated explanations. It is yet to be determined whether epilepsy and its treatment are causing comorbid health conditions or vice versa. There is also a possibility of a common pathogenic mechanism between epilepsy and a comorbid disorder (Seidenberg et al., 2009). Moreover, clinical manifestations of epilepsy include but are not limited to frequent seizures as consequences of excessive excitation, inadequate inhibition of neurons (Ueda et al., 2003), disturbance in mood, sensation, and other cognitive functions (Salpekar, 2016; World Health Organization, 2022). In addition, there is an increased risk of seizure‐related disability and premature mortality in the PWE compared to the general population, with the highest rate (up to 37%) in the LMICs (Moshé et al., 2015).

As mentioned above, most PWE are rural and urban poor of developing countries with failed or weak health‐care infrastructure. The socioeconomic divide also makes it cumbersome for these PWE to seek treatment outside their countries; therefore, most remain “untreated” (Beghi, 2020). In addition to lack of knowledge, there is also an epilepsy‐related stigma among the poor slum dwellers, discouraging them from seeking treatment and worsening their overall health and quality of life (Boling et al., 2018). Although medical complications of epilepsy and its seizures are extensively discussed, personal, economic, academic, and social problems caused by it are often forgotten or overlooked. If a child has epilepsy, there is a high chance that he or she will drop out of school and develop dependent behaviors, hence curbing social and community development (Singh, 2015). It is also worth noting that despite several risk factors associated with epilepsy, the underlying etiology remains unknown in 50% of worldwide cases (World Health Organization, 2022).

The objective of this short review was to evaluate current treatment options and low‐intensity, pulsed‐focused ultrasound (FUS) as a potential new treatment option for epilepsy. Increasing the number of cost‐efficient and effective treatment options not only benefits drug‐resistant patients but is also critically important to narrow the treatment gap in developing countries.

## 2 CURRENT TREATMENT OPTIONS

In the past few decades, substantial progress has been made in the treatment of epilepsy with the development of antiseizure medications (ASMs) and surgical and neuromodulation techniques. Despite the advancement, there is a significant treatment gap in LMICs. It is estimated that 80%–90% of PWE living in developing countries do not have access to appropriate treatment options (Wahab, 2010). Table 1 presents the current treatment options for epilepsy.

**TABLE 1:** Summary of a few current treatment options for epilepsy

**Treatment option**	**Mechanism of action**	**Therapeutic outcomes**	**Adverse effects/major drawback**
Carbamazepine	Alteration of sodium, potassium, or calcium conductance Alters synaptic transmission of neurotransmitters such as GABA and glutamate (Tolou‐Ghamari et al., 2013)	Most effective against partial seizures (Tolou‐Ghamari et al., 2013)	Drowsiness, headaches, migraines, gastrointestinal distress, motor, and coordination disturbances (Tolou‐Ghamari et al., 2013) Can increase several types of seizures (Greenwood, 2000)
Valproic acid	Acts on GABA levels of CNS, inhibits histone deacetylase, and reduces firing of neurons by blocking voltage‐gated ion channels (Rahman & Nguyen, 2022)	Effective against complex partial seizures (Rahman & Nguyen, 2022)	Serious adverse effects include but are not limited to hepatotoxicity, thrombocytopenia, myelosuppression encephalopathy, hypothermia, anaphylaxis (Rahman & Nguyen, 2022)
Phenobarbitone	Acts on GABA_A_ receptor subunits Keeps the chloride ion gates open, and increases the action potential threshold (Kale & Perucca, 2004)	Has broad spectrum efficacy against all types of seizures other than absences (Kale & Perucca, 2004) proven effective for status epilepticus (Lewis & Phenobarbital, 2022)	Some reported adverse events: incoordination, drowsiness, depression, and hepatotoxicity. Reported to have adverse effects on intelligence scores and behavior of children with febrile seizures Excess neuropsychological toxicity compared to other antiseizure medications (Kale & Perucca, 2004; Lewis & Phenobarbital, 2022)
Gabapentin	Exact mechanism is unknown Crosses blood–brain barrier and acts on neurotransmitters (Ziganshina et al., 2017)	Effective against partial seizures (Ziganshina et al., 2017)	Life‐threatening allergic reaction, severe withdrawal symptoms, rebound seizures, angioedema, depression, hepatotoxicity, renal failure, somnolence, sexual dysfunction, dizziness, nausea, vomiting, fatigue, and fever (Ziganshina et al., 2017)
Lamotrigine	Exact mechanism is unknown Selectively binds and inhibits voltage‐gated channels and inhibits presynaptic glutamate and aspartate release (Betchel et al., 2022)	Effective against primary generalized simple and complex partial, and tonic‐clonic seizures (Betchel et al., 2022)	Nausea, vomiting, constipation, headaches, anxiety, visual disturbances, serious rashes, multi‐organ sensitivity, suicidal behavior, and ideations, aseptic meningitis, status epilepticus (Betchel et al., 2022)
Cenobamate	Dual mechanism of action Works by blocking persistent sodium currents (Löscher & Klein, 2021) Increases inhibitory currents via allosterically modifying GABA_A_ receptors (Löscher & Klein, 2021)	Effective in reducing ≥50% frequency of seizures and seizure‐freedom in DRE patients (Lattanzi et al., 2022) Effective against focal‐onset seizures and absence seizures (Löscher & Klein, 2021)	Dizziness, headache, somnolence, diplopia, fatigue (Krauss et al., 2020)
Cannabidiol	Works by blockage of persistent sodium currents (Löscher & Klein, 2021)	Effective against primary generalized tonic‐clonic seizures and focal‐onset seizures in DRE (Löscher & Klein, 2021) Effective for Dravet syndrome and Lennox‐Gastaut syndrome (Löscher & Klein, 2021)	Appetite loss, dizziness, irritability, tremor, ataxia, urinary retention (Geffrey et al., 2015)
Fenfluramine	Works by releasing serotonin (Löscher & Klein, 2021)	Effective against Dravet syndrome in DRE patients (Löscher & Klein, 2021)	Loss of appetite, fever, fatigue, bronchitis, decreased blood glucose level, nasopharyngitis (Nabbout et al., 2020)
Resective surgery focal (temporal and extratemporal resections)	Removes an area of epileptogenesis. Mainly indicated for epilepsy patients with focal seizures who do not respond to well‐chosen and tolerated ASMs therapy consisting of two or more medications (Miller & Hakimian, 2013)	Seizure‐freedom, or long‐term reductions in seizure frequency Reduces risk of complications and comorbidities associated with epilepsy Improved quality of life (Miller & Hakimian, 2013)	Memory, vision, mood, speech, coordination, motor problems, headaches, stroke, bleeding, infections, and deep vein thrombosis (Miller & Hakimian, 2013)
Corpus callosotomy	Splitting the main connection between cerebral hemispheres (Bower et al., 2013)	Effective for both drop seizure and other seizure types (Bower et al., 2013)	Disconnection syndrome Loss of coordination, speech problems including apraxia, and aphasia (Bower et al., 2013)
Hemispherectomy	A rare neurosurgical procedure. Performed mainly in children with seizures arising from a large area on one hemisphere. The diseased half of the brain can be completely or functionally disconnected (Moosa et al., 2013)	Seizure‐freedom, reduction in seizure frequency (Moosa et al., 2013)	Can negatively affect speech, reading abilities, and ambulation (Moosa et al., 2013)

Abbreviation: ASM, antiseizure medications; DRE, drug‐resistant epilepsy; GABA, gamma‐aminobutyric acid.

### 2.1 Antiseizure medications (ASMs)

Although there is a diverse group of effective ASMs, there is still no single ideal first‐line drug for all epilepsy patients (Moshé et al., 2015). Moreover, there is considerable variation in patient response to ASMs depending on the type of seizures, presence of comorbidities, and epilepsy syndrome (Liu et al., 2017). About 30% of patients are estimated to be resistant to ASMs and struggle with devastating comorbidities associated with epilepsy (Wahab, 2010). According to the International League Against Epilepsy, drug‐resistant epilepsy (DRE) is the “failure of adequate trials of 2 tolerated, appropriately chosen and used antiseizure medications schedules (whether as monotherapy or in combination) to achieve sustained seizure freedom” (Kwan et al.). Studies have also shown that in some cases, patients became seizure‐free with certain ASMs; however, later, they developed resistance to the medication (Wahab, 2010). Lattanzi et al. (2022) reported cenobamate to be the most efficient ASM when compared with other third‐generation ASMs for DRE. In two more meta‐analyses, plant‐derived drug cannabidiol and oral fenfluramine showed statistically significant promising results in DRE patients, respectively (Lattanzi et al., 2021; Nabbout et al., 2020).

Furthermore, adverse side effects, withdrawal symptoms, and drug–drug interactions are other concerning issues associated with ASMs. Monotherapy trials involving ASMs face many challenges which include the limited number of participants, effects of dosing on patients’ tolerability, and other potential risks to the patients. Therefore, rare but severe side effects of ASMs might appear after the drug comes to the market (Gilliam, 2003; Wahab, 2010). Antiseizure medication‐induced adverse effects are well known in the literature. These adverse effects can negatively affect the quality of life of epilepsy patients and increase health‐care costs. Some side effects associated with common ASMs such as carbamazepine and valproic acid include drowsiness and dizziness, motor and visual disturbances, hepatotoxicity, and gastrointestinal symptoms (Wahab, 2010; Koliqi et al., 2015). Investigations also show that ASMs only relieve symptoms by suppressing seizures and do not significantly affect epileptogenesis (i.e., the progression of epilepsy) (Wahab, 2010). Moreover, some studies highlight the potentiation and aggravation of certain seizures by ASMs, which lead to increased morbidity and mortality risks. For instance, carbamazepine, gabapentin, phenytoin, phenobarbital, and lamotrigine are all reported to induce or aggravate myoclonic seizures (Wahab, 2010; Senanayake & Román, 1993; Cho & Hong, 2008; Greenwood, 2000).

### 2.2 Epilepsy surgery

When drug therapy fails, surgical approaches such as resection and disconnection are often considered the treatment of choice in selected PWE. However, these treatment options also have their pitfalls and limitations. For instance, a systematic review on complications of focal epilepsy surgery reports that despite several positive outcomes, minor and major complications associated with epilepsy surgeries cannot be denied. According to this review, minor medical complications of resective surgery were seen in about 5.1%. These medical complications included cerebrospinal fluid leak, intracranial or extracranial infection, intracranial hematomas, deep vein thrombosis, pneumonia, aseptic meningitis, and other infections (Hader et al., 2013).

Neurologic, psychiatric, and cognitive disturbances are also not uncommon complications post epilepsy surgery. One investigation describes a pronounced decline in “verbal memory after 19%–59% of dominant temporal resections” (Spencer & Huh, 2008). In another study conducted on 53 patients with multiple subpial transections alone, 10 patients developed significant motor and sensory deficits such as hemiparesis, visual field deficits, and memory decline. Moreover, 37 of 156 patients who had transections with resection also developed similar complications (Spencer & Huh, 2008).

Epilepsy surgery is also a highly technology‐dependent procedure. In addition to high technology, neurosurgeons or professionals who could perform such procedures are more likely to be lacking in resource‐poor and developing countries. Thus, there is a clear need for more alternative, cost‐effective therapies with improved efficacy to benefit all PWE and narrow the treatment gap in developing countries.

### 2.3 Newly emerging therapeutic options for epilepsy

In cases when epilepsy surgery is not suitable for a patient, neuromodulation could be an alternative treatment option. Standard neuromodulation therapies include deep‐brain stimulation, responsive neurostimulation, and vagus nerve stimulation (Lee et al., 2022). These common neuromodulation therapeutic approaches require invasive procedures to implant “pulse generators, electrodes, and battery replacements,” exposing patients to inevitable surgical risks and complications (Lee et al., 2022).

Low‐intensity, pulse‐FUS is also a neuromodulatory but noninvasive technique that can potentially reduce the epileptogenic activity of neurons. This treatment technique has recently drawn extensive attention and has been studied using animal models; however, limited preclinical and clinical studies have been conducted on the safety and efficacy of this technique in humans (Lee et al., 2022).

In a study conducted by Chen et al., the neuromodulatory effects of low‐intensity FUS were investigated in epilepsy‐induced rats via pentylenetetrazol. The study showed that a low‐intensity FUS pulse can be used to significantly suppress abnormal epileptic signal bursts (Chen et al., 2020). Zou et al. also confirmed that ultrasound neuromodulation can have a pronounced effect on reducing the number and duration of seizures in acute epileptic monkeys (Zou et al., 2020). Furthermore, another study has demonstrated that low‐intensity pulsed FUS effectively altered nonlinear dynamics of focal field potential in temporal lobe acute epileptic mice (Li et al., 2019).

In 2020, a pilot study was conducted that included adult patients diagnosed with epilepsy. This study delivered transcranial low‐intensity pulsed ultrasound sonication to the seizure onset zone of the six selected drug‐resistant patients while undergoing stereo‐electroencephalography (SEEG). Simultaneous recordings during treatment and 3 days after treatment showed significant changes in SEEG's spectral power. These changes were observed only in SEEG signals of target electrodes, and no significant changes were observed in nontarget electrodes, thus indicating that FUS has spatial specificity and accuracy compared to other noninvasive neuromodulatory techniques (Lee et al., 2022). Spectral analysis of waveforms from the target electrodes also indicated that only a 10 min sonication was enough to suppress neural activity. Moreover, magnetic resonance imaging was done after receiving the treatment, which also showed no structural lesion and brain edema. No patient showed increased clinical seizures or treatment‐related adverse events (Lee et al., 2022).

The study's results demonstrated that additional studies of low‐intensity FUS's suppressive effects with larger sample cohorts are necessary to assess optimal sonication parameters and highlight the safety and potential clinical effects of this noninvasive therapeutic technique.

## 3 FUTURE RECOMMENDATIONS

Although ASMs, surgical, and neuromodulation approaches have benefited hundreds of thousands of epilepsy patients, there is still a clear need for more treatment options.

No regulatory body has approved FUS as a treatment for epilepsy yet, and a limited number of clinical trials are underway at various health institutions such as Brigham and Women's University, Mayo Clinic, University of Virginia, and Stanford University (Epilepsy. Focused Ultrasound Foundation, 2022). These clinical trials are of significant importance for future clinical application of low‐intensity FUS in PWE worldwide.

Additional studies are also needed to investigate this novel therapy's cost‐effectiveness compared to common neuromodulation techniques. Noninvasive low‐intensity pulsed FUS may offer a cost‐effective approach for treating epilepsy in developing countries. Some of the challenges to introducing this treatment option into developing countries will be delivering the technology and expertise. Although this seems complex, it would be feasible compared to surgical treatment, which requires high technology and years of expertise. With the help of adequate technological resources and educational training, local professionals can be empowered to perform such treatments.

Nevertheless, this novel therapeutic approach has the potential to be added to the list of treatment options for epilepsy. A higher number of cost‐efficient and effective treatment options can benefit more patients and allow them to have a better quality of life. Furthermore, it is extremely important to narrow the treatment gap in developing countries and empower all patients to participate in and contribute to the development of their societies. More efforts should be made to study the efficacy of this novel treatment option so that findings can be used to support continued research and trials.

## CONFLICTS OF INTEREST

The authors have no conflicts of interest to declare.

### PEER REVIEW

The peer review history for this article is available at https://publons.com/publon/10.1002/brb3.2852.

## Data Availability

The datasets generated and/or analyzed during the current study are available from the corresponding author on reasonable request.
